# The relationship between positive and negative stress and posttraumatic growth in university students: the mediating role of resilience

**DOI:** 10.1186/s40359-023-01400-2

**Published:** 2023-10-20

**Authors:** Antonio Serpa-Barrientos, María Luisa Matalinares Calvet, Ana Gloria Díaz Acosta, Ana Cecilia Pareja Fernández, Luisa Hortensia Rivas Díaz, Flor María Ayala Albites, Jacksaint Saintila

**Affiliations:** 1 Departamento Psicología, Universidad Nacional Mayor de San Marcos, Lima, Perú; 2 Universidad Nacional Hermilio Valdizán, Huánuco, Perú; 3 Escuela de Medicina Humana, Universidad Peruana Unión, Carretera Central, Km 19.5, Lima 15, Lima, Perú

**Keywords:** Stress, Resilience, Posttraumatic growth, Multigroup invariance

## Abstract

**Background:**

Information on understanding the mediating role of resilience in the relationship between posttraumatic growth (PTG) and positive and negative stress among students is limited. The objective of this research was to evaluate the mediating role of resilience in the relationship between positive and negative stress and PTG in university students.

**Methods:**

The research was carried out using an associative strategy with an empirical approach and explanatory design, with a sample of 507 participants whose average age was 22.38 years (${\text{S}\text{D}}_{\text{a}\text{g}\text{e}}$= 3.758), the sample was mostly composed of women (70.4%).

**Results:**

The results allow us to describe that resilience plays a complete mediating role in the relationship between negative stress and PTG. On the other hand, mediation was partial when resilience mediated the relationship between positive stress and PTG. In addition, multigroup invariance analyses according to gender and geographic context show that there is no difference in males, females, and the region where they reside.

**Conclusion:**

In conclusion, the hypothesis that resilience fulfills the mediating function is ratified.

## Introduction

In recent years, the world’s population has been in a state of alert and uncertainty as a result of the COVID-19 pandemic [1]; therefore, people, especially university students, generally exposed to academic stress, experienced adverse situations such as loss of family and friends, health problems, work, social confinement, financial imbalance, travel restrictions, limited social interaction, and changes in lifestyle, all of which affected their psychological well-being [2]. Likewise, emotional exhaustion, distress, and deterioration in social and occupational functioning were evidenced, which increased vulnerability to the development of mental health disorders [3, 4] threatening people’s quality of life [5, 6].

The World Health Organization reported 6.3 million deaths from COVID-19, of which 1.3 million were in Asia and 2.7 million in Latin America [7]. Peru recorded the highest mortality rate in the world, reaching 219,597 deaths, which is much higher than the 673.1 mortality rate per 100,000 inhabitants reported by the Ministry of Health (Peru) [8], a circumstance that affected the mental health of people, including university students [9, 10].

In this regard, recent studies revealed that mental health problems such as anxiety were prevalent in 62.7% of young American university students [11]. An additional study conducted in the United States has corroborated that the COVID-19 pandemic has led to increased levels of stress and anxiety (71%) among the population, adding to existing concerns about academic performance, personal health, and that of family members [6]. Similar cases occurred in Chinese university students with acute stress, anxiety, and depressive symptoms (45%) associated with psychosocial factors [12].

The situation described above has also affected Peru, as noted by Llamacuro-Mamani et al., [13] who reported that the COVID-19 pandemic has caused anxiety, depression, and stress in the adolescent and adult population of the country. However, there have been no reports on the positive effects that transformative change could have on young university students, which could strengthen their personal development and increase their self-awareness, a psychological protective factor that would enable them to better face future challenges.

In this sense, post-traumatic growth (PTG) is a positive strategy that increases the capacity to overcome adversity and favors an existential change after facing challenging and shocking events. In addition, it has an impact on the revaluation of the meaning of life, improvement in interpersonal relationships, personal-spiritual strength, greater empathy towards others, a feeling of being “more human” and with greater possibilities of helping others [14], which leads to the reinterpretation of events and builds a new narrative of optimism [15]. There is evidence to suggest that PTG may be directly linked to resilience, which is a dynamic process that is activated after a stressful event and aims to restore balance and proper functioning of the person [16]. In addition, the presence of positive functioning variables such as gratitude, meaning in life, and resilience are indirectly associated with increased PTG during confinement due to COVID-19 [17].

Several studies [18–20] evidenced the relevance of resilience in the process of adaptation to new, critical, and unexpected circumstances or situations to return to normality, which is important in times of pandemics such as COVID-19, due to the daily risks and challenges faced by the population, which favors PTG. Consequently, from what has been described above, it could be summarized that in the face of adverse or traumatic situations (negative experience by COVID-19) experienced by university students, resilience would play a mediating role in the association between negative stress and PTG.

In contrast, Xu et al., [1] concluded that resilience was a moderating variable between state anxiety, trait anxiety, and PTG in medical students exposed to academic stress overload. According to Peng et al. [21] PTG is more frequently observed in male medical students under elevated stress and anxiety, where resilience plays a moderating role. This means that excess load can enhance adaptive capacity in the face of adverse events.

Regarding stress, it is a complex phenomenon that can originate from various factors, whether internal or external, which can be perceived as real or imaginary threats that unbalance the homeostasis of the individual, affecting both the emotional state and physiological indicators [22, 23]. This disruption in homeostasis can trigger a series of cognitive changes that, while commonly presented as challenges in daily life, also possess the potential to be channeled in a positive way to address such challenges. In this way, information processing could be directed in a positive direction, allowing for adaptation and a constructive response to stressful situations, rather than a negative reaction [24, 25]. This perspective contrasts with traditional views that link stress exclusively to negative consequences such as maladjustment, anxiety, depression, and post-traumatic stress disorder [26, 27].

On the other hand, social support and mental health care is positively associated with greater PTG, which enables enhanced resilience [28, 29]. In this regard, Garcia et al. [30] found that reflective and deliberate rumination lead to PTG due to cognitive restructuring, learning, and problem acceptance; and that perceived stress is a more pathological response. For their part, Lee et al. [31] investigated the mediating effects of resilience and its relationship with stress and PTG. The international precedents coincide in affirming that a conditioning factor that is added to the perception of stress is individual differences as a personality resource [32]; to which is added the mediating role of the creation of new meanings in the relationship of stressful events and stress-related growth in university students, through the use of coping strategies can contribute to stress resilience as a predictor of mental health [33, 34]. This shows that when the person interprets having the power to control stressful events, they develop greater adaptive functioning [35].

In conclusion, all studies described above allow us to ask the following question: to what extent does resilience play a mediating role in the relationship between negative stress and PTG in university students? Is there a statistically significant difference according to gender and residence (residents of the capital and province) in the proposed mediating model? Consequently, the aim of this research was to evaluate the mediating role of resilience in the relationship between stress and PTG in university students.

In addition, the following hypotheses are proposed:

General hypothesis: Resilience plays the mediating role between perceived stress (both positive and negative) and posttraumatic growth in Peruvian university students.

Specific hypothesis 1: there is a gender difference in the proposed model, where resilience plays a mediating role between perceived stress (both positive and negative) and post-traumatic growth in Peruvian university students.

Specific hypothesis 2: there is a difference according to geographical context (Lima and other cities) in the proposed model, where resilience plays a mediating role between perceived stress (both positive and negative) and post-traumatic growth in university students.

## Materials and methods

### Study design

According to Sanchez et al. [36], the design of this research is explanatory, since the variables of resilience, perceived stress, and PTG were described, and subsequently the relationship between them was analyzed. Furthermore, the objective of this research is to provide more information in the field of study and compare it with what already exists, which classifies it as basic research.

### Participants

The study population was university students from four regions of Peru: Lima, Huanuco, Junin, and Piura. The type of sampling was non-probabilistic by convenience, where the researchers selected the participants according to accessibility criteria [37]. A total of 507 university students were included in the study, because after validating the data, those records that were not answered in their entirety were eliminated. Data collection began on May 2 and was completed on September 9, 2021.

The 507 participants had an average age of 22.38 years (SD = 3.758), with 70.4% female and the rest male (29.6%). According to the academic cycle, university students of the VII cycle predominated (28%), followed by the V cycle (14.2%). Most of the university students indicated living with both father and mother (53.8%), followed by living only with mother (22.5%). In the context of the COVID-19 pandemic, 53.4% presented symptoms of COVID-19, 35.5% had a positive diagnosis, 33% received the corresponding treatment, and 6.3% required hospitalization. It should be noted that 67.3% lived with someone who presented symptoms of COVID-19 and 52.7% had to personally care for their sick family members (Table 1).

**Table 1:** Sociodemographic description (n = 507)

		f	%
Gender	Female	357	70.4
	Male	150	29.6
Geographical context	Lima	282	55.6
	Huanuco	96	18.9
	Junin	66	13.0
	Piura	63	12.4
Academic cycle	I	10	2.0
	II	13	2.6
	III	49	9.7
	IV	39	7.7
	V	72	14.2
	VI	34	6.7
	VII	142	28.0
	VIII	48	9.5
	IX	37	7.3
	X	21	4.1
	XI	26	5.1
	XII	16	3.2
Currently lives with	Father and mother	273	53.8
	Father only	27	5.3
	Mother only	114	22.5
	Grandparents only	55	10.8
	Uncles only	9	1.8
	Alone	29	5.7
During this pandemic: did I have symptoms of COVID-19?	Yes	296	58.4
	No	211	41.6
During this pandemic: were you diagnosed with COVID-19?	Yes	180	35.5
	No	327	64.5
During this pandemic: did you receive treatment for COVID-19?	Yes	171	33.7
	No	336	66.3
During this pandemic: did you require hospitalization?	Yes	32	6.3
	No	475	93.7
During this pandemic: did anyone living with you have COVID-19?	Yes	341	67.3
	No	166	32.7
During this pandemic: did you care for anyone who had COVID-19?	Yes	267	52.7
	No	240	47.3

Note. f: frequency.

### Variables and measurement instruments

Resilience: The Connor-Davidson instrument (Resilience Scale, CD- RISC 10), whose initial design was constructed by Campbell-Sills and Stein [38], was used. It is a 10-item instrument with a Likert-type response format with five options from *not at all* (0) to *almost always* (4). The CD- RISC was validated in young Peruvian university students by Domínguez-Lara et al. [39]. The internal consistency reliability found for this research was: omega = 0.877 and alpha = 0.876. In this research we evaluated the evidence of validity based on the internal structure of the construct, obtaining results of appropriate fit (χ2 = 105.738, df = 35, CFI = 0.961, TLI = 0.950, SRMR = 0.034). Internal consistency estimates were also obtained with appropriate values (ω = 0.899, α = 0.876), therefore, it is ratified that the scale has appropriate psychometric characteristics for the study sample.

Perceived stress: It was assessed by the Perceived Stress Scale (PSS) designed by Cohen et al. [40] and validated in Peru by Guzmán-Yacaman et al. [41]. It is composed of 13 items and two subscales, one positive and one negative, which allow visualizing which aspects reflect the ability to cope with existing stress (positive items) and which indicators express the inability to self-control (negative items) under stressful situations. Also, this scale consists of five response options for each question on a Likert-type scale: *never, almost never, occasionally, frequently*, and *almost always.* This instrument obtained a reliability omega = 0.808 and alpha = 0.837; while in the dimensions of positive stress (omega = 0.81 and alpha = 0.808) and negative stress (omega and alpha = 0.88).

In this research, it was necessary to evaluate the psychometric properties of this instrument, obtaining evidence of validity based on the internal structure of the construct. An appropriate fit index was obtained (χ2 = 312.094, df = 64, CFI = 0.935, TLI = 0.905, SRMR = 0.0679); internal consistency estimates were also obtained with appropriate values (ω_stress−_ = 0.808, ω_stress+_ = 0.880, α_stress−_ = 0.776, α_stress+_ = 0.842), ratifying that the scale has adequate psychometric characteristics for the study sample.

Post-traumatic growth: This variable was studied with the Posttraumatic Growth Inventory (PTGI), which was developed by Tedeschi et al. [14]. This instrument evaluates the benefits and positive consequences that people perceive after experiencing an adverse situation and is comprised of 21 items answered on a 6-point Likert scale. (0 *= I did not experience this change as a result of my crisis*, 1 = *a very small*, 2 = *a small degree*, 3 = *a moderate degree*, 4 = *a great degree*, and 5 = *I experienced this change to a very great degree as a result of my crisis*). The reliability found for this study was: omega = 0.897 and alpha = 0.896. For this study, it was also necessary to evaluate the psychometric properties of this instrument, obtaining evidence of validity based on the internal structure of the construct, with the results of an appropriate fit index (χ2 = 276.499, df = 65, CFI = 0.921, TLI = 0.905, SRMR = 0.049). Similarly, internal consistency estimates were obtained with appropriate values (ω = 0.901, ω = 0.896), confirming that the scale has appropriate psychometric properties.

### Procedure

The research protocol was reviewed and approved by the Research Ethics Committee of the Universidad Nacional Mayor de San Marcos (N° RR: 05753-R-21). Subsequently, authorization was requested from the directors of the professional schools of the selected universities. After obtaining a favorable response to evaluate university students, informed consent was obtained from all participants. In addition, the objectives and aims of this research were explained. Finally, the survey was applied.

### Statistical analysis

After data collection was completed, the information was systematized and validated using Microsoft Excel, version 2016. Then, statistical analysis was carried out using different types of software such as SPSS version 26 [42] and JAMOVI version 2.2. [43]. Furthermore, the extreme scores of the data were evaluated, for which it was necessary to analyze using skewness and kurtosis with values between ± 1.5 [44–46] that justify the univariate normality. On the other hand, multivariate normality analysis was obtained using Mardia’s distance coefficient (G^2^) with values ≤ 5.0 [47].

Subsequently, it was necessary to obtain the psychometric properties of the three instruments, such as validity based on internal structure through Confirmatory Factor Analysis (CFA) and reliability through the internal consistency method using the alpha and omega coefficient. For this purpose, R Studio software was used specifically employing the Lavaan package [48].

Regarding the hypothesis testing of the research, AMOS software was used to evaluate the mediating role of resilience in the association between stress and PTG under the following parameters: Complete mediation is considered when the indirect measures (Stress± → Resilience → PTG) are statistically significant, while the direct influence (Stress± → PTG) is not. On the other hand, partial mediation is when indirect and direct measures are statistically significant. Finally, null mediation occurs when only the direct measure is statistically significant and the other indirect measures are not (Ato y Vallejos, 2015). Finally, regarding the comparison of the specific hypotheses, the Chi-square (χ2) and degrees of freedom (df) parameters were used as an overall measure, taking into account that the ratio (χ2/df) is statistically significant (p < .05). This will allow us to interpret whether there are significant differences according to gender and geographical context of the participants.

## Results

With respect to the descriptive analysis, the results allow us to describe that the univariate normality is justified because the values were found in the parameters described as adequate (± 1.5) except for item 1 of the resilience scale, where the values of skewness and kurtosis were found slightly higher than the allowed threshold. Regarding the multivariate normality analysis, the result obtained allows us to describe that the data did not follow a multivariate distribution (G^2^ = 54.510), because the coefficient was higher than the expected parameter (G^2^ ≤ 5.0). Consequently, the CFA was performed using the weighted least squares mean and variance adjusted (WLSMV) estimator because the data does not meet the assumption of multivariate normality and is ordinal in nature.

**Fig. 1:**
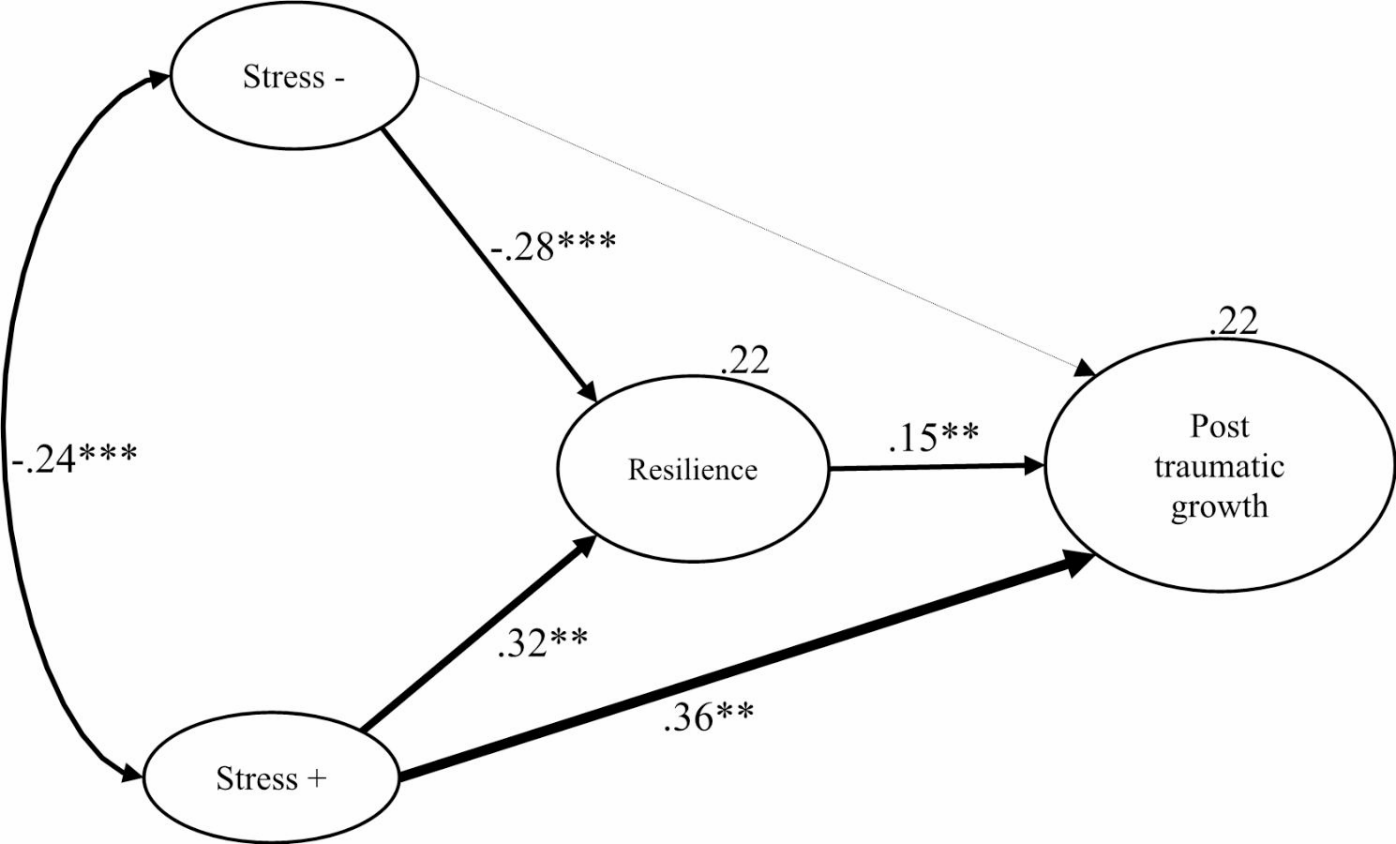
Graphical representation of the pathway of the study variables (n = 507) Note. Model fit indices: χ2/df = 2.864, CFI = 0.957, TLI = 0.947, SRMR = 0.054, RMSEA = 0.061 CI 90% [0.052, 0.070]

According to the results obtained (see Table 2; Fig. 1), it can be inferred that the variable resilience would be fulfilling the mediating function in the association between stress and PTG in Peruvian university students. Regarding the parameters of the direct effect of negative stress completely mediated by resilience on PTG, the results are statistically significant (Stress → Resilience → PTG, β = − 0.04, p < .002). This means that negative stress experienced by university students has a statistically significant influence on their ability to exhibit resilient behavior, however, it is an inverse influence (Stress → Resilience, β = − 0.28, p < .001). That is, when university students experience higher levels of negative stress, they tend to exhibit lower resilience, which in turn is related to lower PTG. These findings suggest that negative stress may have a negative impact on students’ ability to develop resilience, which, in turn, may affect their ability to experience PTG. Furthermore, we found that resilience exerts a significant influence on PTG, supported by robust estimates (Resilience → PTG, β = 0.15, p < .001). However, the direct relationship between negative stress and PTG was not statistically significant (Stress- → PTG, β = − 0.09, p = .043). Additionally, we observed that the connection between positive stress and PTG, mediated by resilience, is partially effective (Stress+ → Resilience → PTG, β = 0.05, p < .001). This suggests that stress, in its positive aspect, may have a direct influence on PTG, regardless of whether resilience mediates this relationship or not. In essence, it is observed that the PTG is explained by 22% of the total variability, which reflects the correlation between stress and PTG in the context of this study. These results indicate that high levels of negative stress reduce levels of resilience, and low levels of resilience predict low levels of PTG. Although high levels of positive stress increase levels of resilience, and this in turn leads to higher levels of post-traumatic growth. This would support the idea of the need to change negative stress states for positive ones due to their protective effect on PTG, in an attempt to counteract the risk factor that implies high levels of negative stress.

**Table 2:** Parameters indirect, direct, and total effect

				95% CI				
Type	Effect	Estimator	SD	LL	UL	β	Z	P
Indirect	Stress- → Resilience → PTG	-0.415	0.134	-0.677	-0.153	-0.04	-3.103	0.002
	Stress+ → Resilience → PTG	0.482	0.151	0.186	0.778	0.05	3.188	0.001
Component	Stress- → Resilience	-1.235	0.179	-1.586	-0.884	-0.28	-6.899	< 0.001
	Resilience → PTG	0.336	0.097	0.147	0.525	0.15	3.475	< 0.001
	Stress+ → Resilience	1.435	0.179	1.084	1.786	0.32	8.015	< 0.001
Direct	Stress- → PTG	-0.826	0.408	-1.625	-0.027	-0.09	-2.027	0.043
	Stress+ → PTG	3.435	0.414	2.625	4.246	0.36	8.304	< 0.001
Total	Stress- → PTG	-1.241	0.395	-2.015	-0.468	-0.13	-3.145	0.002
	Stress+ → PTG	3.918	0.395	3.144	4.691	0.41	9.924	< 0.001

Note. SD: Standard deviation. Confidence intervals computed with method: Standard (Delta method). Betas are completely standardized effect sizes. LL: Lower Limit, UL: Upper Limit, β: structural regression coefficient, Z: critical ratio, p: statistical significance.

As for the multigroup comparison (measurement invariance) according to the contrast variable (gender), the working hypothesis was not satisfactorily corroborated. This result allows us to specify that the implication of positive and negative stress mediated by resilience on the PTG is invariant when compared between male and female university students in five cities in the Peruvian context. Therefore, the comparison for each parameter β is detailed in Table 3 (e.g., the incidence of negative stress on resilience for both genders is statistically significant [β_male_ = 0.272**, β_female_ = 0,397***, Δβ = -0.125, p > .05], but invariant).

**Table 3:** Multigroup invariance according to gender of the proposed model

	Multigroup invariance	χ2	DF	Δβ	Δp	Interpretation
Overall test	Without restrictions	2132.289	74,325			
	Constrained	2140.737	8653			
	Difference	8.448	65,672			
	P-value	1.000				The p-value of the chi-square difference test is not significant; interpret local tests with caution.
	Route name	β Male	β Female	Δβ	Δp	Interpretation
Local tests	F3 → F1.	0.272**	0,397***	-0.125	1.000	No difference.
	F4 → F1.	-0.155	-0.394***	0.239	1.000	The negative relationship between F1 and F4 is only significant for Female.
	F3 → F2.	0.378***	0.371***	0.007	1.000	No difference.
	F4 → F2.	-0.173†	-0.075	-0.098	1.000	The negative relationship between F2 and F4 is only significant for Male.
	F1 → F2.	0.046	0.262***	-0.216	1.000	The positive relationship between F2 and F1 is only significant for Female.

Note. Indicators of statistical significance: † p < 0,100, * p < 0,050, ** p < 0,010, *** p < 0,001, F1: resilience, F2: post-traumatic growth, F3: negative stress, F4: positive stress, “Multigroup Analysis”, AMOS Plugin. StatWiki de Gaskination.

The results indicate that the proposed model shows no significant differences in terms of geographical context (place of residence), which means that the relationship between the model variables (resilience, PTG, and negative stress and positive stress) is consistent both in Lima (capital city) and in the provinces. Statistically significant differences were not found in the regression coefficients between the geographic routes for any of the relationships analyzed. This suggests that the model is generalizable and that the variables maintain similar relationships in different geographical contexts, which strengthens the reliability of the study’s conclusions in both locations (Table 4).

**Table 4:** Multigroup invariance according to the geographical context of the proposed model

	Multigroup invariance	χ2	DF	Δβ	Δp	Interpretation
Overall test	Without restrictions	2167.135	99,648			
	Constrained	2170.54	74,801			
	Difference	3.405	24,847			
	P-value	1.000				The p-value of the chi-square difference test is not significant; interpret local tests with caution.
	Route name	β Lima (capital)	β Province	Δβ	Δp	Interpretation
Local tests	F3 → F1.	0.371***	0.293**	0.078	1.000	No difference.
	F4 → F1.	-0.301***	-0.366**	0.066	1.000	No difference.
	F3 → F2.	0.339***	0.508***	-0.169	1.000	No difference.
	F4 → F2.	-0.102†	-0.173†	0.071	1.000	No difference.
	F1 → F2.	0.130*	0.237*	-0.108	1.000	No difference.

Nota. Indicators of statistical significance: † p < 0,100, * p < 0,050, ** p < 0,010, *** p < 0,001, F1: resilience, F2: post-traumatic growth, F3: negative stress, F4: positive stress, “Multigroup Analysis”, AMOS Plugin. StatWiki de Gaskination

## Discussion

The COVID-19 pandemic brought with it multiple consequences for both physical and mental health, including somatic symptoms, changes in thinking, feelings, and behavior related to excessive preoccupation with health. Therefore, from a clinical and health psychology perspective, various characteristics and implications of the COVID-19 pandemic have been analyzed, including the consequences of elevated stress on individuals, especially university students.

Consequently, the purpose of this research was to evaluate the mediating role of resilience in the relationship between positive and negative stress with PTG in Peruvian university students. The results allow us to describe that resilience acts as a complete mediator in the relationship between negative stress and PTG. Similarly, it was verified that resilience serves as a partial mediator in the relationship between positive stress and PTG. These results suggest that, in the face of a negative stress event, the presence of resilient behaviors is necessary to generate changes and allow the revaluation of the meaning of life. It was also found that this mediating role does not differ according to gender or geographic context. However, when analyzing the dynamics with positive stress, notable differences linked to gender were identified.

One of the main aspects of this research is to contribute to the understanding of the theoretical basis of PTG, building on previous research that has confirmed the important role of PTG, negative as well as positive stress, and resilience in adaptation. In this context, it is essential to bear in mind that a conditioning factor that adds to the perception of stress is the existence of individual differences, as highlighted in previous studies [32]. These individual differences may include personality resources that influence how individuals experience and cope with stress. Furthermore, the mediation assessment implies that resilience acts as a link in the chain between negative stress and PTG, indicating that negative stress can decrease resilience, but not completely eliminate resilient behavior. It is important to highlight that this fraction of resilient behavior opens the possibility of generating changes in thinking, feelings, and behavior to redefine strategies that allow valuing the meaning of life and, therefore, experiencing PTG. These findings are in line with previous research suggesting that negative stress may decrease resilience, whereas resilience may increase PTG [49, 50]. Through these findings, this research seeks to provide a solid basis for understanding how negative and positive stress, resilience, and PTG interact and contribute to the adaptation of individuals, considering individual differences as well as the mediating role of resilience. Additionally, these results can be used to support the adaptation of models that attempt to clarify and specify the factors contributing to PTG in different groups according to sex and place of residence, providing valuable information on mental health promotion in stressful contexts such as the devastating COVID-19 pandemic.

It is important to highlight that there is another perspective in which positive stress can also be a key factor in strengthening resilience. This type of stress, known as eustress, can provide challenges and opportunities that can help university students develop coping, adaptive and personal growth skills [51]. In situations of eustress, individuals often discover internal resources they did not know they had, which contributes to their resilience and recovery capacity [52]. Therefore, although negative stress can have an impact on resilience, positive stress also plays an important role in its strengthening and continued development. In addition, gender differences in the perception and experience of stress, resilience, and PTG suggest that men and women may have unique approaches to coping with stressful situations. Female university students appear to associate positive stress with their resilience, indicating that they may benefit from interventions aimed at strengthening this resilience in times of stress. On the other hand, the negative relationship between positive stress and resilience in women suggests that they face additional challenges in situations of positive stress, highlighting the need for specific coping strategies for them. Finally, the significant relationship between negative stress and PTG in men may reflect differences in how genders experience and process stressful events, which has important implications for care and treatment. Overall, these gender differences emphasize the importance of considering individual diversity in mental health and the need to tailor intervention and support strategies according to an individual’s gender.

### Limitations

The limitations of this research are described below. First, the study variables (stress, resilience, and PTG) were measured with self-report and participation was voluntary, therefore, the associations between the three variables could have been influenced by common method variance [53]. Second, the study was cross-sectional, which would imply that the results have less scope in terms of predictive validity and the inferences obtained are exclusive to the study sample. Finally, more variables (e.g., coping styles, emotional regulation, self-efficacy) that are likely to explain PTG were not included. Consequently, future research should consider these constructs to verify whether these variables influence the association between stress and PTG.

However, despite the limitations described, this research contributes to the understanding of the association between stress, resilience, and PTG. Specifically, the mediating role of resilience in the relationship between negative stress and PTG, suggesting that resilience can be considered as a priority target for intervention by mental health specialists to improve coping skills in university students under stressful situations. Furthermore, the results of this study have relevant implications for public health because they draw attention to the fact that resilience is a crucial factor in the development of PTG. Furthermore, it has clinical relevance because it broadens and provides new insights into the influence of stress and resilience on PTG. Therefore, these findings have obvious implications for health centers, organizations, and public health agencies interested in designing, implementing, and executing education programs related to positive mental health prevention and promotion in university students. Moreover, variations in how each gender perceives and experiences stress, resilience, and PTG underscore the importance of recognizing the uniqueness of each individual in the mental health arena. This observation reinforces the premise that intervention and assistance tactics should be shaped and personalized by considering the gender particularities of each individual.

## Conclusion

The findings indicate that resilience plays a mediating role in the relationship between negative and positive stress with PTG in the population analyzed. It is important to note that this mediation does not vary according to gender and geographic environment. This implies that, regardless of gender and place of origin, the perception and impact of resilience as an intermediary element remains constant, with no significant differences between the different groups.

## Data Availability

The data sets generated to support the findings of this study are not publicly available due to certain legal restrictions established by the Research Ethics Committee of the Universidad Nacional Mayor de San Marcos, but can be requested from the corresponding author.
